# The LRR-RLK Protein HSL3 Regulates Stomatal Closure and the Drought Stress Response by Modulating Hydrogen Peroxide Homeostasis

**DOI:** 10.3389/fpls.2020.548034

**Published:** 2020-11-27

**Authors:** Xuan-shan Liu, Chao-chao Liang, Shu-guo Hou, Xin Wang, Dong-hua Chen, Jian-lin Shen, Wei Zhang, Mei Wang

**Affiliations:** ^1^Key Laboratory of Plant Development and Environmental Adaption Biology, Ministry of Education, School of Life Sciences, Shandong University, Qingdao, China; ^2^School of Municipal and Environmental Engineering, Shandong Jianzhu University, Jinan, China; ^3^State Key Laboratory of Plant Genomics, Institute of Genetics and Developmental Biology, The Innovative Academy of Seed Design, Chinese Academy of Sciences, Beijing, China

**Keywords:** *HSL3*, drought stress, abscisic acid, hydrogen peroxide, guard cell

## Abstract

Guard cells shrink in response to drought stress and abscisic acid (ABA) signaling, thereby reducing stomatal aperture. Hydrogen peroxide (H_2_O_2_) is an important signaling molecule acting to induce stomatal closure. As yet, the molecular basis of control over the level of H_2_O_2_ in the guard cells remains largely unknown. Here, the leucine-rich repeat (LRR)—receptor-like kinase (RLK) protein HSL3 has been shown to have the ability to negatively regulate stomatal closure by modulating the level of H_2_O_2_ in the guard cells. *HSL3* was markedly up-regulated by treating plants with either ABA or H_2_O_2_, as well as by dehydration. In the loss-of-function *hsl3* mutant, both stomatal closure and the activation of anion currents proved to be hypersensitive to ABA treatment, and the mutant was more tolerant than the wild type to moisture deficit; the overexpression of *HSL3* had the opposite effect. In the *hsl3* mutant, the transcription of NADPH oxidase gene *RbohF* involved in H_2_O_2_ production showed marked up-regulation, as well as the level of catalase activity was weakly inducible by ABA, allowing H_2_O_2_ to accumulate in the guard cells. HSL3 was concluded to participate in the regulation of the response to moisture deficit through ABA-induced stomatal closure triggered by the accumulation of H_2_O_2_ in the guard cells.

## Introduction

Drought stress imposes a major restriction on crop productivity. An important part of the plant response to this stress is the control imposed on stomatal closure. In *Arabidopsis thaliana*, stomatal closure is affected by a change in the volume of the pair of guard cells that surround each stomata (Kwak et al., 2008). This stomatal movement is mediated by the phytohormone abscisic acid (ABA), which accumulates in response to drought stress. ABA exerts this effect through its activation of anion channel currents and inhibition of inward K^+^ channel currents (Schroeder et al., 2001a). A common event associated with the response of plants to a diversity of abiotic stresses (including drought stress) is the accumulation of reactive oxygen species (ROS) such as O_2_^–^, H_2_O_2_, and HO^–^ (Foyer and Noctor, 2000). High concentrations of ROS damage plant cells, whereas at low concentrations, ROS function as signaling molecules, controlling plant growth and the stress response (Pastori and Foyer, 2002; Apel and Hirt, 2004). In particular, the guard cells use H_2_O_2_ as a signaling molecule within the ABA signaling pathway (Bright et al., 2006; Jo et al., 2006; Li et al., 2017). H_2_O_2_ produced by ABA activates inward calcium channels, which is essential for Ca^2+^ influx to guard cells (Pei et al., 2000; Kwak et al., 2003; Islam et al., 2019). Cytosolic calcium increase is essential to ABA activation of S-type anion channels and suppression of inward-rectifying K^+^ channels in *Arabidopsis* guard cells, leading to the efflux of K^+^ and stomatal closure (Berkowitz et al., 2000; Schroeder et al., 2001a).

In mutants unable to produce the enzyme NADPH oxidase, ABA-promoted H_2_O_2_ production and stomatal closure are both impaired, resulting in a hypersensitivity to drought stress (Kwak et al., 2003). The ROS-scavenging enzymes catalase, ascorbate peroxidase, and glutathione peroxidase have evolved as a major mechanism preventing the overaccumulation of H_2_O_2_ (Mhamdi et al., 2010). In rice, the protein DST regulates stomatal movement by controlling the synthesis of a precursor of the peroxidase used to neutralize H_2_O_2_ in the guard cells (Huang et al., 2009), while it has emerged that H_2_O_2_ can also be scavenged by glutathione peroxidase (Miao et al., 2006). The identity of the genes regulating the expression of H_2_O_2_-scavenging enzymes in the guard cells remains largely unknown, as does the mechanism used to control the local level of H_2_O_2_.

Receptor-like kinases (RLKs) control plant growth, development, and stress responses (Stone et al., 1998; Wan et al., 2008). The *A. thaliana* genome encodes at least 610 RLKs, among which 216 have been classified as leucine-rich repeat (LRR) RLKs (Shiu and Bleecker, 2001; Hwang et al., 2011). As yet, approximately only 20 of these LRR-RLKs have been matched with a ligand (Butenko et al., 2009). Many LRR RLKs have been proved to regulate responses to environmental stress signals. For example, the protein HPCA1 acts as a cell surface sensor of H_2_O_2_, mediating the H_2_O_2_-induced activation of Ca^2+^ channels in the guard cells (Wu et al., 2020). Moreover, the RLK GHR1 modulates the H_2_O_2_-ABA signaling pathway involved in the process of stomatal closure (Hua et al., 2012). And the ABA-inducible protein RPK1 has been reported to regulate the *A. thaliana* response to drought stress (Hong et al., 1997; Osakabe et al., 2005, 2010). However, the underlying molecular mechanisms remain unclear. The largest group of LRR-RLKs (subclass XI) includes the HAESA (HAE) and HAESA-LIKE2 (HSL2) receptor proteins. Either HAE or HSL2 is required to direct cell separation during floral organ abscission in *A. thaliana* via its ligand INFLORESCENCE DEFICIENT IN ABSCISSION (IDA) (Butenko et al., 2003; Cho et al., 2008; Stenvik et al., 2008). Our previous study showed that IDL6-HAE/HSL2 promotes the susceptibility to Pst DC3000 and the pectin digestion in *A. thaliana* leaves (Wang et al., 2017). Plant stomata, which consist of a pair of guard cells, serve as the major sites to defend against not only pathogen attack but also drought stress. The present report focuses on the function of HSL3 in stomatal movement. Our current findings indicate that HSL3 negatively regulates the plant’s tolerance of drought stress and controls H_2_O_2_-mediated stomatal closure in response to ABA signaling.

## Materials and Methods

### Growth Conditions and Plant Materials

*A. thaliana* ecotypes Col-2 or Col-0 represented the wild type (WT). The T-DNA insertion mutant *hsl3-1* (Salk_207895) was created in a Col-0 background, whereas *hsl3-2* (WISCDSLOX450B04) was created in a Col-2 background. Seed of all four genotypes was obtained from ABRC (abrc.osu.edu/). Lines overexpressing *HSL3* (OE#5, OE#10) were generated by amplifying 3,212 nucleotides coding sequence from *Arabidopsis* sscDNA using the primer pair Gate-HSL3-F/-R (Table 1), inserting the resulting amplicon into pB2GW7.0 to generate the construct p*35S:HSL3*. The construct was introduced into *Agrobacterium tumefaciens* strain GV3101 and transformed into *A. thaliana* Col-0 using the floral dip method (Clough and Bent, 1998).

**TABLE 1:** PCR primer sequences (shown 5′–3′).

**Mutant identification**	
HSL3-LP/-RP	TGCTCTGGTATTCCTCCAGTG/ATCCCAGATGTTTT ATTCGGG
T-DNA-BP	AACGTCCGCAATGTGTTATTAAGTTGTC
**RT-PCR**	
HSL3-F/-R	ATGACTCGTTTACCCTTACCTTTCC/TTATACAAAACCT AAATCTTCATCTTCTACC
ACTIN2-F/-R	TCTTCTTCCGCTCTTTCTTTCC/TCTTACAATTTCCCGC TCTGC
**qRT-PCR**	
qCAT1-F/-R	TCAAATGCCTGTCGGATGAG/GAAGAGATTCCACTGCG GATAG
qCAT2-F/-R	CTTTACACCAGAGAGGCAAGAA/TCAGCCTGAGACCA GTAAGA
qCAT3-F/-R	CTCCAGTCTCCAACAACATCTC/GGATCCTCTCTCTG GTGAAATTAG
qTUA6-F/R	CTGGTATGTTGGTGAGGGTATG/CAGCACCGACCTCT TCATAAT
qUBQ10-F/R	CGGATCAGCAGAGGCTTATTT/GGGTGGATTCCTTCTG GATATTG
qHSL3-F/-R	GTATGTTCTGCGCCAACAAG/CTTGTCCTTCGACCCG ATAAA
qACTIN2-F/-R	GGTAACATTGTGCTCAGTGGTGG/AACGACCTTAATCT TCATGCTGC
qAPX1-F/-R	GTCCATTCGGAACAATGAGGTTTGAC/GTGGGCACCA GATAAAGCGACAAT
qRBOHD-F/-R	TCAACAACATGAAAGGTCC/CTAGAAGTTCTCTTTGTGG
qRBOHF-F/-R	CAGCAACCGCCATTAATG/CATCGAACAGTTCCAATGC
qSOD1-F/-R	AAGTAACCAAAGAGAGACGAAGCA/TGAAAAAGATAGT CCCCGTAACAC
**Plasmid construction**	
Gate-HSL3-F/-R	GGGGACAAGTTTGTACAAAAAAGCAGGCTTCATGACT CGTTTACCCTTACCTTTC/GGGGACCACTTTGTACAAG AAAGCTGGGTCTTATACAAAACCTAAATCTTCATCTTCT

Seeds were surface-sterilized by immersion in 75% ethanol for 5 min, air-dried on a sterile filter paper, and laid on 0.7% wt/vol agar containing half-strength (Murashige and Skoog, 1962) (1/2 MS) medium. After holding in the dark for 3 days at 4°C, the seeds were transferred to a growth chamber [approximately 70% relative humidity, 100 μmol m^–2^ s^–1^ light (14 h light/10 h dark cycles), 22°C ± 1°C/16°C ± 4°C day/night cycles] for further growth. After ∼8 days, the seedlings were potted into a 2:1 mixture of soil and vermiculite.

### Phylogenetic Tree Analysis and Prediction of Phosphorylation Sites

Nucleotide and deduced amino acid sequences were obtained from GenBank, and an unrooted phylogenetic tree was generated by the neighbor-joining method using MEGA X software (Kumar et al., 2018). PhosPhAt 4.0^1^ was used to predict the phosphorylation sites in HSL3.

### Reverse Transcription–Polymerase Chain Reaction and Quantitative Real-Time PCR

Template for reverse transcription–polymerase chain reactions (RT-PCRs) was prepared from total RNA extracted from either whole seedlings or leaves using the TRIzol reagent (DBI Bioscience, Shanghai, China), treated with RNase-free DNase I (Promega, Madison, WI, United States), and reverse-transcribed based on an oligo (dT) primer and Super Script*R*^TM^ III Reverse Transcriptase (Invitrogen, Carlsbad, CA, United States), following protocols provided by the manufacturer. The quantitative real-time (qRT)–PCR method was applied to profile *HSL3* transcription used as template sscDNA derived from RNA extracted and processed as above from 2 week-old seedlings exposed to either 10 μM ABA, 5 mM CaCl_2_, 100 μM H_2_O_2_, 50 μM SNP [sodium nitroprusside, an nitric oxide (NO) donor], or dehydration stress. The reactions were based on FastStart Universal SYBR Green Master reagent (Roche, Basel, Switzerland). The chosen reference sequences were *AtACTIN2* (*At3g18780*), *AtTUA6* (*At4g14960*), and *AtUBQ10* (*At4g05320*), and the analytical platform was the CFX96 Touch^TM^ Real-Time PCR Detection System (Bio-Rad, Hercules, CA, United States). The primer sequences required for both the RT-PCR and qRT-PCR assays were designed using Primer Premier v5.0 software^2^ and are listed in Table 1.

### Whole-Plant Drought Stress and Leaf Water Loss Bioassays

For drought stress assay, the seedlings were grown under well-watered conditions for 4 weeks, and then the plants were deprived of water for 3 weeks. After withholding water, the plants were rehydrated over 3 days before photographing. For water loss measurement, rosette leaves were detached from 4 week-old plants (at least three replicates per treatment per genotype) and laid on dry filter paper in the light. The leaves were weighed over a period of 3 h at various time points. The water loss rate was calculated on the basis of the initial fresh weight of the plants.

### Measurement of Stomatal Closure

Measurements of stomatal aperture were made following a method described in detail elsewhere (Zou et al., 2010). Rosette leaves were harvested from 4 week-old plants and floated for 2.5 h in closure solution [20 mM KCl, 1 mM CaCl_2_, 5 mM MES-KOH (pH 6.15)] in the light with their adaxial surface facing upward. Subsequently, the closure solution was supplemented with one of 10 μM ABA, 100 μM H_2_O_2_, 5 mM Ca^2+^, 50 μM SNP, 20 mM 3-amino-1,2,4-triazole (3-AT, an inhibitor of catalase), 0.3 mM DIDS (4,4’-diisothiocyanatostilbene-2,2’-disulfonic acid), 20 μM NPPB (5-nitro2,3-phenylpropylaminobenzoic acid), 20 μM diphenyleneiodonium chloride (DPI, an NADPH oxidase inhibitor), 100 U mL^–1^ catalase (a ROS scavenger), or absolute ethanol as a control in which the leaves were incubated for a further 2.5 h. Abaxial epidermal stripes were then peeled away, and the stomata imaged by light microscopy. The images were used to estimate stomatal dimensions with the help of ImageJ v1.37 software (imagej.nih.gov/ij/). SigmaPlot v11.0 (sigmaplot.softonic.com) software was used to draw the charts.

### Net Efflux of Cl^–^

The net efflux of Cl^–^ was measured in 4 week-old plants using a Non-invasive Microtest Technique (NMT100; Younger United States) described elsewhere (Xu et al., 2006; Sun et al., 2009). Briefly, epidermal leaf strips were attached to the base of a Petri dish and incubated for 2 h in 0.05 mM ABA, 0.1 mM CaCl_2_, and 0.3 mM KCl (pH 6.0) under light (100 μmol m^–2^ s^–1^). A control set of samples was incubated under the same conditions except in the absence of ABA. The strips were rinsed three times in the ABA-free solution, and the dishes were refilled with the ABA-free solution. The Cl^–^ flux microsensor was about 10 μm above the guard cell. Every guard cell was recorded for 5 min and repeated for 3–6 times. Data were acquired from at least six guard cells. NMT100 Series (Younger USA LLC, Amherst, United States) was used. The Cl^–^ selective microelectrodes with an external tip (1 ± 0.5 μm in diameter) were manufactured and silanized with tributylchlorosilane. The resulting images were quantified using SigmaPlot v11.0 (sigmaplot.softonic.com) and imFluxes V2.0 (Younger USA LLC, Amherst, United States) software.

### Assay for H_2_O_2_ and ROS

The 3,3′-diaminobenzidine (DAB) uptake method (Thordal-Christensen et al., 1997; Guan and Scandalios, 2000) was used to monitor the production of H_2_O_2_ from excised leaves. Fully expanded leaves were harvested and divided into a test group and a control group. The latter leaves were incubated in distilled water and the former ones in 10 μM ABA for 30 min in the dark at 28°C. The leaf samples were then immersed in a pH 3.8 solution of 1 mg mL^–1^ DAB (Sigma–Aldrich, St. Louis, United States) for 8 h in the dark at 28°C, after which the leaves were boiled for 10 min in 80% (vol/vol) ethanol. Prior to being photographed, the leaves were held at 4°C in 80% (vol/vol) ethanol. ROS production in guard cells was detected using the cell-permeant 2’,7’-dichlorodihydrofluorescein diacetate (H2DCFDA) reagent (Miao et al., 2006). Abaxial epidermis peels were divided into two groups and floated for 2.5 h in 1 mM CaCl_2_, 20 mM KCl, and 5 mM MES-KOH (pH 6.15) to induce stomatal opening. The solution for the test group of samples was made to ABA final concentration of 10 μM and was held for a further 2.5 h. After this step, the samples were exposed to 0.1% wt/vol H2DCFDA for 20 min in the dark, rinsed to remove any excess dye, and imaged by confocal laser-scanning microscopy, using an excitation wavelength of 488 nm. ImageJ v.1.37 software was used to quantify fluorescence intensity (Shen et al., 2017).

### Measurement of Catalase Activity

A CAT (catalase) assay kit purchased from Beyotime (Shanghai, China) was used to detect CAT activity in 4 week-old rosette leaves, following the manufacturer’s protocol. Briefly, leaf lysate was added into the mixed buffer containing a certain amount of H_2_O_2_. The reaction was stopped by adding the Stop Solution 3 min later. The residual H_2_O_2_ was assayed by adding a chromogenic substrate by spectrophometrically (520 nm) tracking its oxidation into the red compound (N-(4-antipyryl)-3-chloro-5-sulfonate-p-benzoquinonemonoimine).

### Pathogen Inoculations and Quantification

*Pst*DC3118 inoculation assay was performed as described (Zipfel et al., 2004). Briefly, the bacterial suspension (2 × 10^5^ colony-forming units/mL) was syringe infiltrated into leaves of 5 week-old *A. thaliana* plants. Three days later, the leaves were ground with 200 μL sterile water and then diluted for 3,000–8,000 multiples to coat plate. The colony numbers were counted 3 days later.

## Results

### Transcriptional Profiling of HSL3 and the Site of Activity of Its Promoter

**FIGURE 1:**
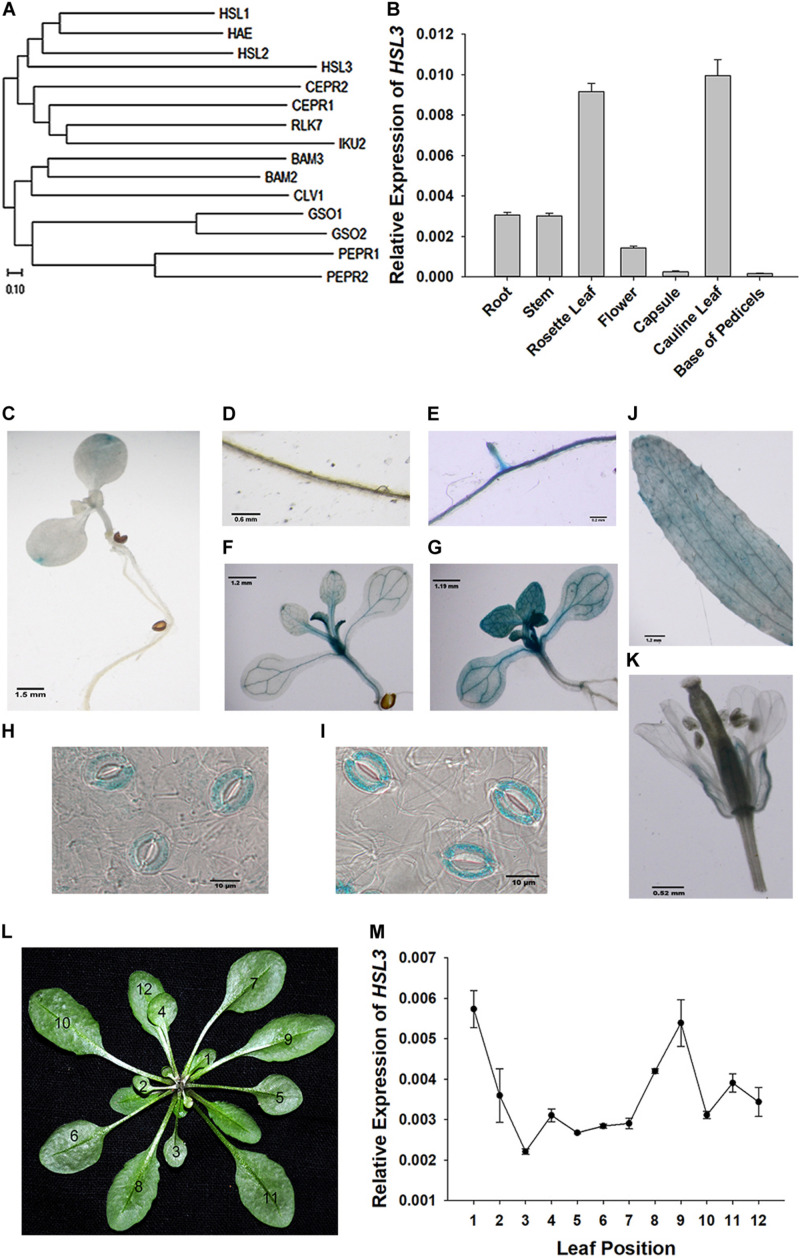
Characterization of the *HSL3* sequence. **(A)** Phylogeny of HSL3, inferred using the neighbor-joining algorithm. An optimal tree with a total branch length of 5.92 is shown. **(B)** The transcription of *HSL3* in *A. thaliana* based on qRT-PCR assays. Values are shown as mean ± SE (*n* = 3). **(C–K)** Expression of the p*HSL3:GUS* transgene identifies the sites where *HSL3* is expressed. The images represent plants **(C**,**D**,**F**,**J**,**K**,**H)** not exposed to ABA and **(E**,**G**,**I)** exposed to 10 μM ABA. **(L)** Rosette leaves developed by 4 week-old plants: the numbers refer to the age of each leaf (12: youngest, 1: oldest). **(M)** *HSL3* transcription during the development of the rosette. Values are shown as mean ± SE (*n* = 3). Relative expression levels of *HSL*3 were normalized with the gene expression of *ACT2*, *TUA6*, and *UBQ10* in **(B,L)**.

**FIGURE 2:**
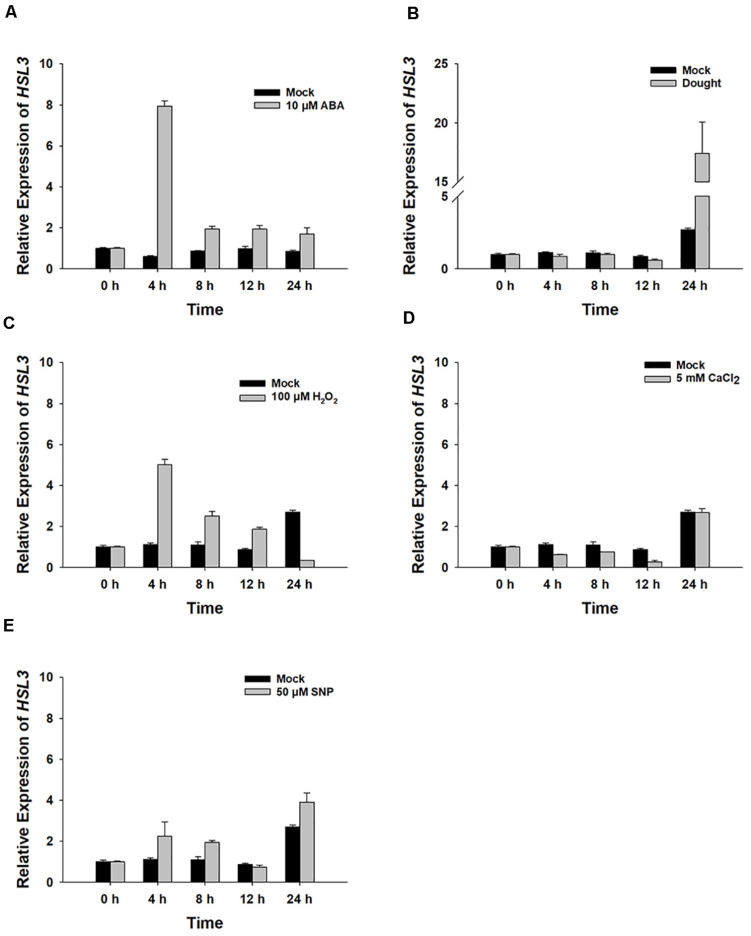
The *HSL3* transcriptional response of 8 day-old WT seedlings upon exposure for between 0 and 24 h to **(A)** 10 μM ABA, **(B)** drought stress, **(C)** 100 μM H_2_O_2_, **(D)** 5 mM CaCl_2_, and **(E)** 50 μM SNP. Values are shown as mean ± SE (*n* = 3). *ACT2*, *TUA6*, and *UBQ10* were used as an internal control for qRT-PCR. The relative expression levels of *HSL3* at 0 h were set at 1.0.

The gene *At5g25930*, which is inducible by pathogen infection, encodes an LRR-RLK protein harboring an extracellular LRR, a transmembrane domain, and an intracellular kinase domain (Hou et al., 2014). A phylogenetic analysis showed its product to fall within the HAE/HSL2 family (Figure 1A). Because PhosPhAt analysis suggested that the gene’s translation product included three of the five HAE/HSL2 phosphorylation sites in its kinase domain (Supplementary Figure S1), it has been denoted *HSL3*. qRT-PCR analysis showed that *HSL3* transcript was abundant in the root and stem and highly abundant in the rosette and cauline leaves of plants grown under non-stressed conditions (Figure 1B). In contrast, transcript of both *HAE* and *HSL2*, both of which share extensive homology with *HSL3*, was restricted to the base of the pedicel and to inflorescence branches (Stø et al., 2015). To further determine the expression pattern of *HSL3*, *HSL3* promoter fragment was fused with the b-glucuronidase (GUS) gene, and the transgenic *A. thaliana* carrying HSL3pro:GUS was analyzed. In the absence of stress signal, *HSL3* was detected on the tip of cotyledon of 9 day-old seedling, in the vascular tissue of leaves of 13 day-old seedling, in the cauline leave and flower as well (Figures 1C,D,F,J,K). However, in the presence of ABA, GUS activity was highly abundant throughout the root, stem, and leaves (Figures 1E,G). *HSL3* was also detected in the guard cell of 4 week-old plant especially in the presence of ABA (Figures 1H,I). Transcriptional profiling of rosette leaves harvested from 4 week-old plants revealed differences in the intensity *HSL3* transcription between young, mature, and senescing leaves (Figures 1L,M). Exposure to both ABA and drought stress up-regulated *HSL3* (Figures 2A,B). Treatment with either H_2_O_2_ or NO (provided by SNP), downstream signals of ABA pathway (Pei et al., 2000; Jo et al., 2006; Zou et al., 2015), also induced an increase in the abundance of *HSL3* transcript (Figures 2C–E). All these data indicated that *HSL3* transcript was most abundant in the leaves, with particularly plentiful expression in the guard cells and that ABA could induce the expression of *HSL3*, suggesting a potential role of *HSL3* in the regulation of ABA mediated stomatal movements and drought stress response.

### HSL3 Negatively Regulates ABA-Induced Stomatal Closure

**FIGURE 3:**
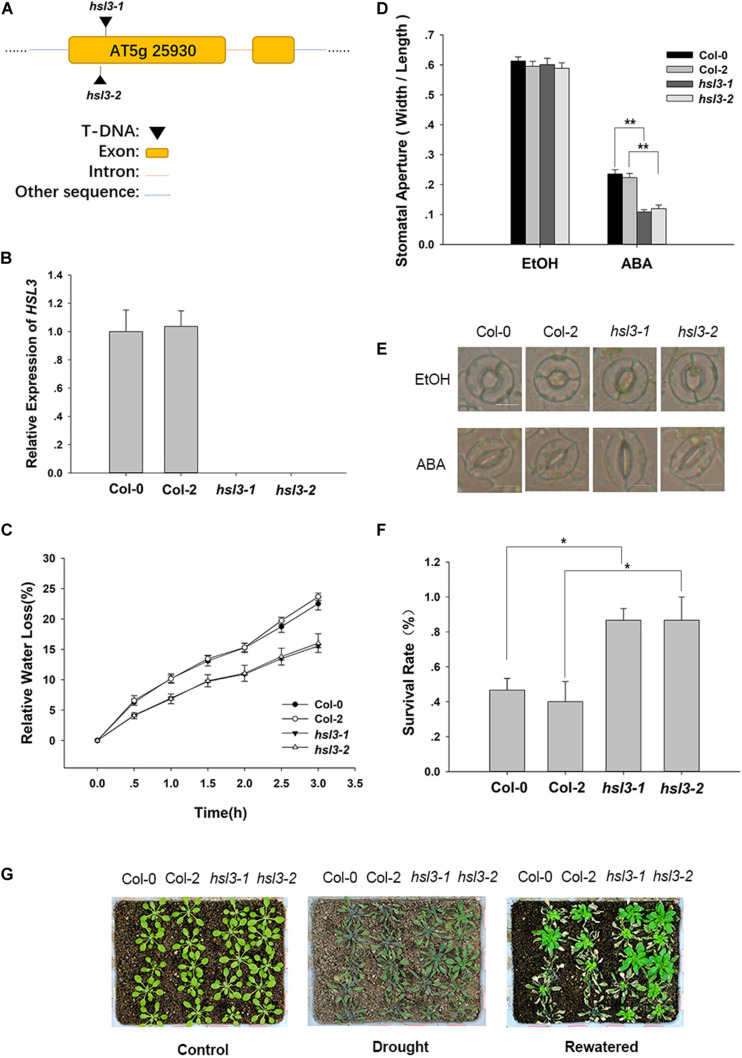
The *hsl3* mutants are more sensitive to ABA with respect to stomatal closure and more drought tolerant. **(A)** The structure of *HSL3* and the site of the T-DNA insertion in the *hsl3-1* and *hsl3-2* mutants. **(B)** The abundance of *HSL3* transcript in Col-0, Col-2, *hsl3-1*, and *hsl3-2* plants. *Actin2*, *TUA6*, and *UBQ10* were used as reference sequences in the qRT-PCRs. Values are shown as mean ± SE (*n* = 3). **(C)** The rate of water loss from detached leaves of Col-0, Col-2, *hsl3-1*, and *hsl3-2* plants. **(D**,**E)** Treatment with 10 μM ABA promotes stomatal closure in Col-0, Col-2, *hsl3-1*, and *hsl3-2* plants. EtOH: controls in which the plants were treated with just ethanol. In **(D)**, values are shown as mean ± SE (*n* = 40); *, **: mean performance differed significantly (*P* < 0.05, 0.01) from WT. Data are shown as mean ± SE (*n* = 40). Bar in **(E)**: 10 μm. **(F)** The survival rate of Col-0, Col-2, *hsl3-1*, and *hsl3-2* plants grown under conditions of moisture stress. Data are shown as mean ± SE (*n* = 15). **(G)** The appearance of Col-0, Col-2, *hsl3-1*, and *hsl3-2* plants grown under conditions of moisture stress.

**FIGURE 4:**
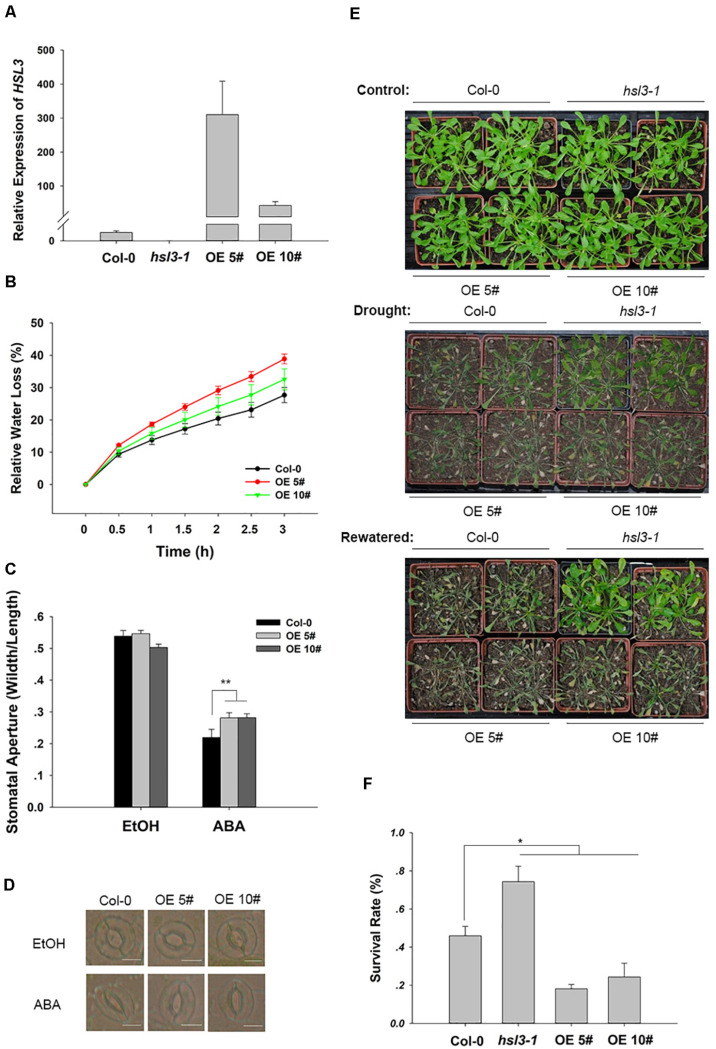
*HSL3* overexpression resulted in ABA hyposensitivity of stomatal closure and decreased drought tolerance. **(A)** The abundance of *HSL3* transcript in WT, *hsl3*, and two overexpression line (OE#5 and OE#10) plants. *Actin2*, *TUA6*, and *UBQ10* were used as reference sequences in the qRT-PCRs. Values are shown as mean ± SE (*n* = 3). **(B)** Time courses of water loss from detached leaves of Col-0, *hsl3*, and the overexpression plants. **(C,D)** The promotive effect of 10 μM ABA on stomatal closure in Col-0 and the overexpression plants. ABA was dissolved in ethanol (EtOH). EtOH was used as control. The experiments were repeated three times; *,**: mean performance differed significantly (*P* < 0.05, 0.01) from WT, respectively. In **(C)**, values are shown as mean ± SE (*n* = 40); bar in **(D)**: 10 μm. **(E)** The phenotype of wild type, *hsl3*, and the overexpression plants grown under drought stress conditions. **(F)** The survival rate of above plants growing under drought stress conditions. Each experiment was repeated three times with similar results.

To further confirm the physiological role of HSL3 in plant stress response, two independent loss-of-function mutants (*hsl3-1* and *hsl3-2*) (Figures 3A,B) and overexpressors (OE#5 and OE#10) (Figure 4A) were obtained. A detached leaf assay showed that the leaves of both *hsl3* mutants experienced a lower rate of water loss than those of WT plants, whereas those of the OE lines experienced a higher rate (Figures 3C, 4B). The whole plant assay of tolerance to drought stress confirmed the superiority of the mutant over WT seedlings (Figures 3F,G). Stomatal aperture in plants not exposed to ABA was independent of genotype, but in ABA-treated plants, it was increased in the OE line relative to WT plants and was diminished in the mutants (Figures 3D,E, 4C,D). The suggestion is that HSL3 negatively regulates both ABA-mediated stomatal closure and the response to drought stress.

### HSL3 Negatively Regulates ABA-Activated Guard Cell Anion Efflux

**FIGURE 5:**
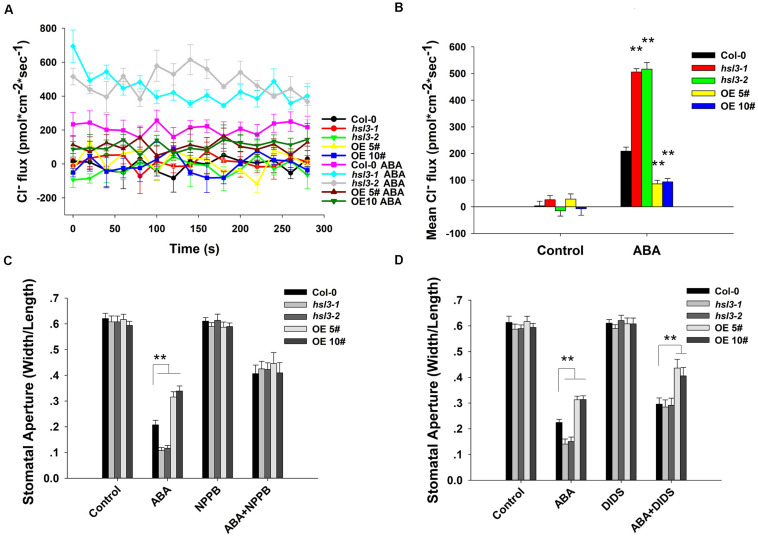
The effect of ABA treatment on anion flux in the guard cells. **(A)** A continuous read-out over 5 min of the Cl^–^ flux in the guard cells of 4 week-old WT, *hsl3-1*, *hsl3-2*, and two overexpression line (OE#5 and OE#10) plants following a 2 h incubation in the presence/absence of 10 μM ABA. **(B)** Mean Cl^–^ fluxes derived from the traces shown in **(A)**. The error bars represent mean ± SE (*n* = 6). ^∗∗^indicates significant difference from one another at *P* < 0.01. **(C**,**D)** Stomatal closure assay in the presence/absence of **(C)** 20 μM NPPB and/or 10 μM ABA, **(D)** 0.3 mM DIDS and/or 10 μM ABA of WT (Col-0), *hsl3-1*, *hsl3-2*, and two overexpression line (OE#5 and OE#10) plants. Both of the experiments were repeated three times. ^∗∗^indicates significant difference from one another at *P* < 0.01. All data represent mean ± SE (*n* = 40).

Anion efflux channels have been shown to play an important role during stomatal closure (Vahisalu et al., 2008; Geiger et al., 2009; Lee et al., 2009); it was of interest to test whether the efflux of Cl^–^ differed in guard cells of wild-type and *hsl3* mutants. In plants not exposed to ABA, the size of the anion efflux of the *hsl3* mutant guard cells was indistinguishable from that in WT plants. In contrast, in plants exposed to ABA for 2 h, substantially more anion efflux were observed in the mutants’ guard cells and less efflux in the overexpressors’ (Figures 5A,B). When slow anion efflux channels were blocked by NPPB and DIDS (Schroeder et al., 1993), the differences in stomatal aperture between WT, mutant, and OE plants were greatly reduced (Figures 5C,D). The data indicated therefore that HSL3 negatively regulated the ABA-induced activation of anion efflux during stomatal closure.

### HSL3 Negatively Regulates H_2_O_2_-Mediated Stomatal Closure

**FIGURE 6:**
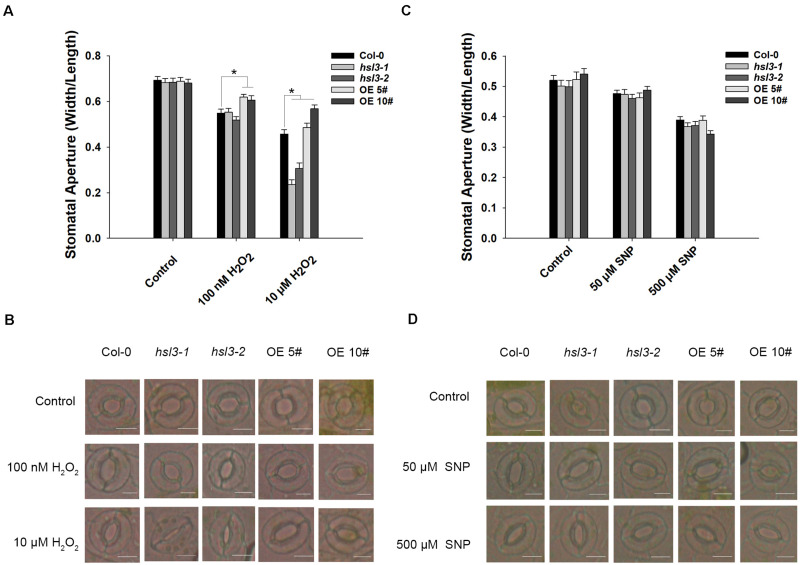
HSL3 negatively regulates H_2_O_2_-induced stomatal closure. The effect of **(A**,**B)** H_2_O_2_ treatment, **(C**,**D)** SNP treatment on stomatal closure in WT, *hsl3*, and two overexpression line (OE#5 and OE#10) plants. Data in **(A**,**C)** are shown as mean ± SE (n = 40). *mean performance differed significantly (*P* < 0.05) from WT. Bar in **(B**,**D)**: 10 μm.

ABA signal causes H_2_O_2_ accumulation and induces the activation of Ca^2+^ channels and slow anion efflux channels in the guard cells (Hamilton et al., 2000; Pei et al., 2000; Schroeder et al., 2001a; Vahisalu et al., 2008). H_2_O_2_ and NO are both the downstream signals in ABA signaling pathway, and NO is dependent on H_2_O_2_ formation (Jo et al., 2006). Next, WT, *hsl3* mutants and OE plants were then compared with respect to the effect of H_2_O_2_ and NO treatment on stomatal closure. In plants not exposed to H_2_O_2_, stomatal aperture was indistinguishable between the three genotypes, but when the plants were treated with 0.1 μM H_2_O_2_, stomatal aperture in the OE plants was notably greater than in WT plants, whereas the difference between WT and the *hsl3* mutants was only marginal. Exposure to 10 μM H_2_O_2_, however, induced a significant reduction in stomatal aperture in the *hsl3* mutant compared to WT, whereas it was markedly larger in the OE plants (Figures 6A,B). The response to ABA was similar (Supplementary Figure S2). However, treatment with SNP, an NO donor, had no differential effect on stomatal aperture (Figures 6C,D). These results suggested that H_2_O_2_ signal was involved in HSL3 mediated stomatal closure.

### HSL3 Negatively Modulates H_2_O_2_ Accumulation in Leaves

**FIGURE 7:**
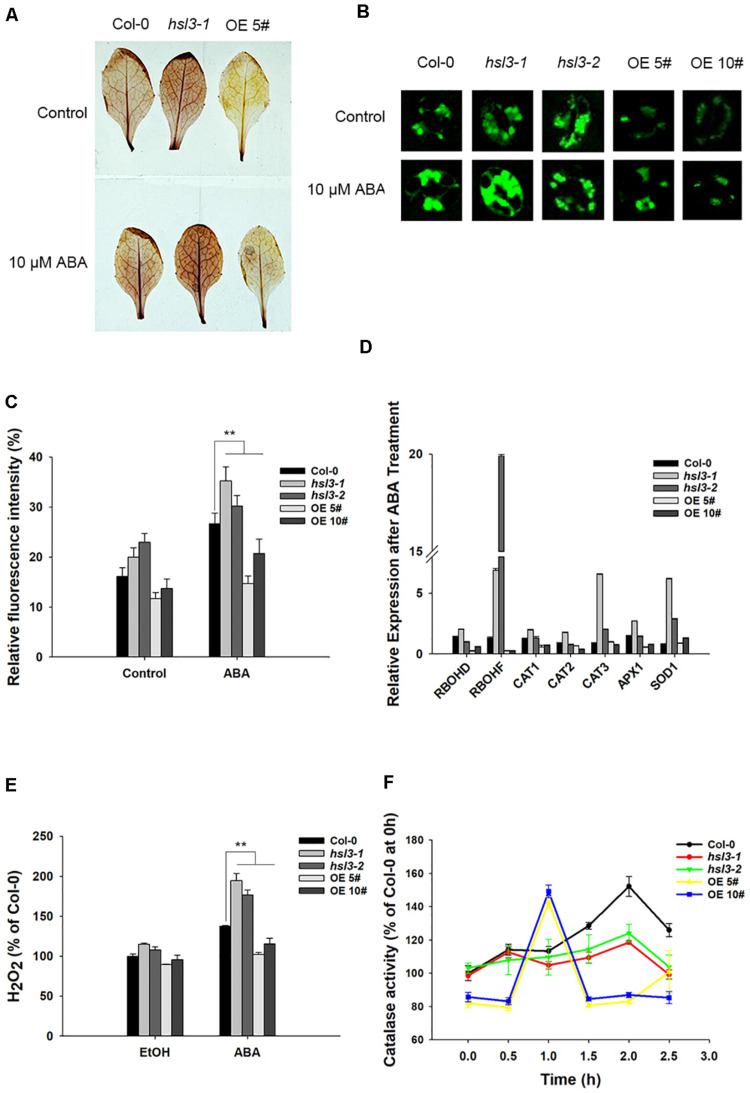
HSL3 negatively regulates the accumulation of H_2_O_2_. **(A)** The ABA-induced production of H_2_O_2_ in detached leaves of WT, *hsl3*, and overexpression line OE#5 plants, as assayed by DAB staining. **(B)** Visualization of H_2_O_2_ production in the guard cells of WT, *hsl3*, and overexpression line OE#5 plants either exposed or not exposed (control) to ABA, as assayed by H2DCFDA staining. **(C)** Quantification of the fluorescence signal shown in **(B)**. Data are shown as mean ± SE (*n* = 30). **: mean performance differed significantly (*P* < 0.01) from WT. **(D)** Transcription of genes encoding ROS homeostasis–related proteins in WT, *hsl3*, and overexpression line OE#5 plants in response to a 2.5 h exposure to 10 μM ABA. Values are shown as mean ± SE (*n* = 3). **(E)** The H_2_O_2_ content of rosette leaves of WT, *hsl3*, and overexpression line OE#5 plants either exposed (ABA) or not exposed (EtOH) to 10 μM ABA. Data are shown as mean ± SE (*n* = 3). ** mean performance differed significantly (*P* < 0.01) from WT. **(F)** Catalase activity in WT, *hsl3*, and overexpression line OE#5 plants exposed to 10 μM ABA. Data are shown as mean ± SE (*n* = 3).

H_2_O_2_ is an important signal molecule that induces stomatal closure (Apel and Hirt, 2004); next, we would like to know whether H_2_O_2_ levels show any difference between WT, *hsl3* mutant, and OE plants. 3’,3’-DAB staining was employed to assay the production of H_2_O_2_ in leaves of the *hsl3* and OE plants. The mutant’s leaves accumulated more H_2_O_2_ and the OEs less H_2_O_2_ than WT leaves, whether or not the plants had been exposed to ABA (Figure 7A). When the ROS indicator H2DCF-DA was used to quantify the total ROS in the guard cells, it was revealed that in plants not exposed to ABA, more ROS was generated in the mutant plants’ guard cells than in WT plant ones, whereas the opposite was the case for the OE plants’ guard cells (Figures 7B,C). Treatment with ABA raised the guard cell H_2_O_2_ content in all three genotypes, but a significantly higher quantity was accumulated in the mutant plants’ guard cells than in WT plant ones and lower quantity in the OE plants’ guard cells (Figures 7B,C,E). Thus, HSL3 appeared to negatively impact ROS production, whereas ABA-mediated stomatal closure in *hsl3* mutant plants was probably due to the overaccumulation of H_2_O_2_ in their guard cells.

Transcriptional profiling, as enabled by qRT-PCR, showed that in *hsl3* mutant plants, genes encoding the NADPH oxidases RbohD and RbohF (which regulate ROS production and participate in guard cell ABA signaling) responded positively to ABA treatment, whereas in OE plants, they responded negatively (Figure 7D). The genes encoding the ROS-scavenging enzymes superoxide dismutase (*SOD1*) and catalase (*CAT3*) were both significantly induced by the ABA treatment in the mutants (Figure 7D). A comparison of leaf catalase activities showed that in WT plants, the ABA treatment resulted in a marked rise, starting 1.5 h after the treatment and peaking after 2 h; in OE plants, the induction began earlier (within 1 h), but the peak level reached was lower; finally, in *hsl3* mutant plants, there was not any detectable induction (Figure 7F). The results indicated that HSL3 negatively regulated H_2_O_2_ accumulation in leaves from two aspects, transcription of *RbohF* and catalase activity, which may lead to the change in drought tolerance.

### HSL3-Mediated Stomatal Closure Depends on H_2_O_2_ Accumulation in Guard Cell

**FIGURE 8:**
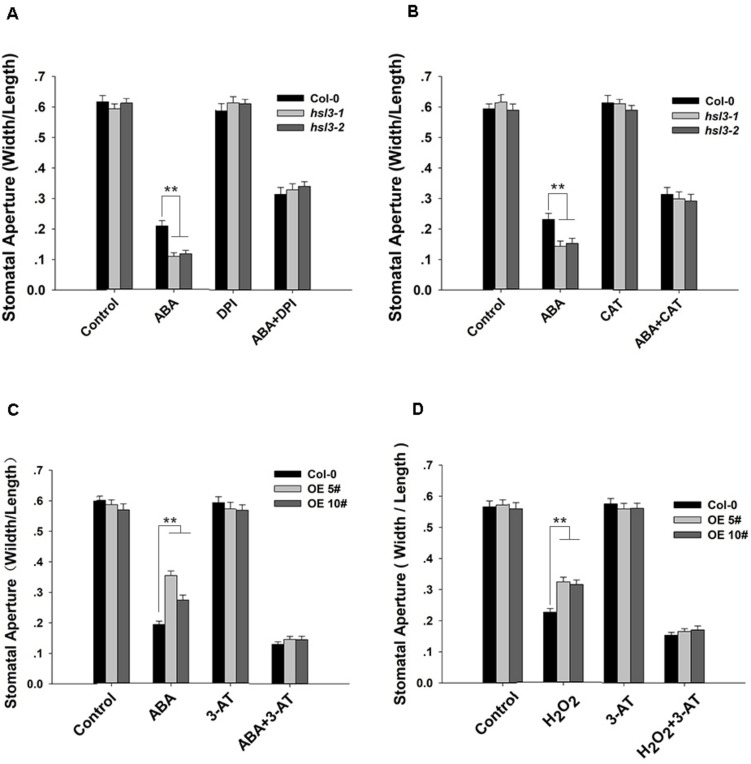
H_2_O_2_ homeostasis is involved in HSL3-mediated stomatal closure. **(A**,**B)** Stomatal closure assay on plants (Col-0 and the mutants *hsl3-1*, *hsl3-2*) exposed to **(A)** ABA and/or DPI, **(B)** ABA and/or CAT. Control: no additive in the solution. **(C**,**D)** Stomatal closure assay on plants [Col-0 and two overexpression lines (OE#5 and OE#10)] exposed to **(C)** ABA and/or 3-AT, **(D)** H_2_O_2_ and/or 3-AT. Control: no additive in the solution. Data are shown as mean ± SE (*n* = 40). ** means performance differed significantly (*P* < 0.01) from WT.

To further verify the role of NADPH oxidase and catalase in HSL3-mediated stomatal closure, we conducted stomatal closure assay with ABA or H_2_O_2_ together with DPI (an NADPH oxidase inhibitor), CAT (a ROS scavenger), or 3-AT (an inhibitor of catalase) treatments. In the mutants, ABA-induced stomatal closure was strongly inhibited when DPI or CAT was added to the closure solution (Figures 8A,B). ABA promoted stomatal closure in WT and to a lesser extent in OE plants. The addition 3-AT had no detectable effect on stomatal closure; however, when either ABA or H_2_O_2_ was combined with 3-AT, the difference in stomatal aperture between WT and OE plants was abolished (Figures 8C,D). Pharmacologically, these results confirmed that HSL3-mediated stomatal closure depended on H_2_O_2_ accumulation in guard cell.

## Discussion

It has been well documented that plant cells respond to drought stress by a rapidly accumulation of ABA. Within the guard cells, ABA stimulates the production of ROS, thereby activating plasma membrane calcium channels and elevating Ca^2+^ current-mediated S-type anion channels, finally driving stomatal closure (Hamilton et al., 2000; Pei et al., 2000; Schroeder et al., 2001a, b). Here the product of *HSL3*, a member of the HAE/HSL2 family, was identified as a regulator of cellular ROS (especially H_2_O_2_) content. Through its modulation of guard cell anion efflux, it has the capacity to negatively regulate ABA-induced stomatal closure and the response to drought stress.

ABA participates in a wide range of developmental events, including inhibition of germination and root growth (Chen et al., 2006; Gao et al., 2007), stomatal closure (Hunt et al., 2003), and regulation of gene expression (Xin et al., 2005). In plants experiencing drought stress, the OST1 protein, a component of the ABA signaling pathway, promotes the cellular content of H_2_O_2_ by its activation of *RbohD* and *RbohF*, genes that both encode an NADPH oxidase (Kwak et al., 2003; Sirichandra et al., 2009). The burst of H_2_O_2_ mediates NO generation and, in turn, activates mitogen-activated protein kinase (MAPK) cascades, and eventually activates slow-type anion channel and causes stomatal closure in leaves (Jiang and Zhang, 2002; Hu et al., 2005; Zhang et al., 2007). Earlier studies also indicate that H_2_O_2_ activates Ca^2+^ channels (Pei et al., 2000) to regulate stomatal movement through Ca^2+^-dependent proteins (Mori et al., 2006; Geiger et al., 2010, 2011; Zou et al., 2010) or MAPKs (Jammes et al., 2009). The cytosolic Ca^2+^ accumulation induced by H_2_O_2_ also activates the anion current through SLAC1 (Pei et al., 2000; Mori et al., 2006; Geiger et al., 2011). Therefore, H_2_O_2_, which acts as a second messenger in ABA signaling pathway, could amplify ABA signal when H_2_O_2_ is accumulated in guard cells. In our study, while the H_2_O_2_ content of WT plants rose significantly in response to exogenously supplied ABA, the increase was more pronounced in the *hsl3* mutant (Figure 7E). At the same time, the mutant exhibited a more marked up-regulation of *RbohF* and a fall in catalase activity (Figures 7D,F). The loss of function of *HSL3* resulted in the stomata becoming more sensitive to an ABA treatment and increased the level of anion efflux in the plasma membrane (Figures 3, 5). In contrast, anion efflux in the *HSL3* overexpressors was significantly lower than that in WT plants (Figure 4). A potential scenario here is that the absence of HSL3 permits a greater accumulation of H_2_O_2_, thereby activating the anion channels within the guard cells and thus accelerating stomatal closure. It has been shown that a rice zinc finger transcription factor, DST, regulates H_2_O_2_-induced stomatal closure by an ABA-independent pathway (Huang et al., 2009). *HSL3* was strongly induced by the exogenous ABA treatment (Figure 2A), and there was a clear difference between WT and the *hsl3* mutant plants with respect to both their stomatal movement and their anion efflux (Figures 3, 5). The indication is therefore that HSL3 regulates H_2_O_2_-induced stomatal closure by acting within an ABA-dependent pathway.

While ROS act as secondary messengers during the regulation of root growth, stomatal movement, germination, and stress response (Kwak et al., 2003; Mittler et al., 2004; Miller et al., 2010), their excessive accumulation imposes oxidative stress and, if prolonged, induces cellular damage and even death. As a result, their level has to be stringently regulated. The most important agents involved in the neutralization of ROS are catalases, ascorbate peroxidases, various types of peroxiredoxins, glutathione/thioredoxin peroxidases, and glutathione S-transferases (Willekens et al., 1995; Asada, 1999; Wagner et al., 2002; Dietz, 2003; Mittler et al., 2004; Iqbal et al., 2006). Here, the ABA-induced production of H_2_O_2_ was greater in the *hsl3* guard cells than in WT ones (Figure 7), implying an impairment to the machinery used to maintain H_2_O_2_ homeostasis. For H_2_O_2_, catalase is one of the most important enzymes to maintain ROS homeostasis. CAT1 and CAT2 are responsible for at least 95% of the catalase activity present in the *A. thaliana* leaf (Mhamdi et al., 2010). However, in this study, only *CAT3* was strongly induced along with *RbohF* and *SOD1* in mutant leaves (Figure 7D), which means little impact was caused by ABA on H_2_O_2_ scavenging at the transcriptional level. The strong up-regulation in the *hsl3* mutant of *RbohF*, which was induced by exogenous ABA treatment, and its reduced level of catalase activity probably act together to limit the plant’s control over H_2_O_2_ accumulation (Figures 7D,F). While *HSL3*, which appears to encode an RLK, was induced by H_2_O_2_, it remains to be ascertained whether HSL3 can sense variation in the local H_2_O_2_ concentration.

The stomata represent a major route of entry for a number of pathogens; meanwhile, ROS, notably H_2_O_2_ and O_2_^–^, had been implicated in the plant’s defense response (Asselbergh et al., 2008). Experimentally, *hsl3* mutant plants appeared to be more resistant to infection by *Pseudomonas syringae* than WT ones, whereas *HSL3* overexpressors were more susceptible (Supplementary Figure S3). The likelihood was that the characteristic reduced openness of the *hsl3* mutant’s stomata was directly responsible for this pleiotropic effect, because it hindered the pathogen entry into the host. However, the HSL3 functional relevance between biotic and abiotic stresses still needs to be elucidated in the future. *HAE* and *HSL2* share extensive homology with *HSL3*; however, *HSL2* hardly expresses in the leaves, with its transcript restricted to the base of the pedicel and to inflorescence branches (Stø et al., 2015). Moreover, the *hls2* mutant did not show visible phenotype in stomatal closure and drought stress response (data not shown); therefore, HSL3 acts differently with HSL2 in guard cell ABA signaling. Recent studies have implicated the role of small signaling peptides in the regulation of stomatal aperture (Qu et al., 2019). For example, *Arabidopsis*-secreted peptide PIP1 was found to regulate not only immune response but also stomatal closure through its receptor, RLK7 (Hou et al., 2014; Shen et al., 2020). The peptides for the HSL3 remain unclear; however, HSL3 perception is likely to involve IDA/IDL peptides. Expression analyses show that *IDL6* and *IDL7* genes respond to a variety of biotic and abiotic stresses (Vie et al., 2015). *IDL6* promotes the *Arabidopsis* susceptibility to Pst DC3000 through its receptor HAE and HSL2 (Wang et al., 2017). Furthermore, *IDL7* acts as a negative modulator of stress-induced ROS responses in *Arabidopsis* (Vie et al., 2017). In a recent study, NbenIDA1 and NbenIDA2 (IDA-like family members of *Nicotiana benthamiana*) homologs were implicated in the responses to drought stress (Ventimilla et al., 2020). Thus, it is possible that IDA/IDL-HSL3 modulates the drought stress response by controlling stomatal aperture in *Arabidopsis* leaves, and it is also interesting to dissect whether HSL3 is needed in IDL7-mediated ROS responses.

**FIGURE 9:**
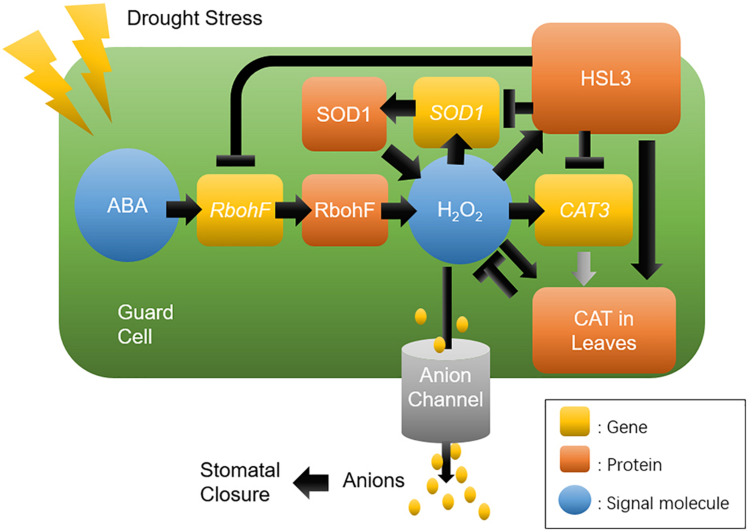
A proposed model for the role of HSL3 in the regulation of stomatal closure and drought tolerance. Drought stress induces the production of ABA and H_2_O_2_, which induces *HSL3* transcription, in turn modulating the expression of ROS homeostasis–related genes *RbohF*, *SOD1*, and *CAT3*; On the other hand, it also modulates the catalase activity in leaves. The net effect is to maintain a low level of H_2_O_2_, which influences anion channel activity in the plasma membrane. Ultimately, tolerance to drought stress depends on effective stomatal closure.

In summary, a proposed model for the function of HSL3 in the regulation of ROS homeostasis, and thus its control over stomatal movement and the plant’s drought stress response, is presented in Figure 9. Drought stress promotes the accumulation of ABA and H_2_O_2_ and up-regulates *HSL3*. The heightened presence of HSL3 maintains a balance between ROS production and removal. Thus, in the *HSL3* loss-of*-*function mutant, H_2_O_2_ overaccumulates, altering transmembrane anion efflux in the guard cells and resulting in stomatal closure and an enhanced tolerance of drought stress. The interesting possibility is that the engineering of crop plants to express *HSL3* at a lower level could enable them to be grown in regions where the availability of soil moisture is restricted.

## Data Availability Statement

The raw data supporting the conclusions of this article will be made available by the authors, without undue reservation.

## Author Contributions

WZ supervised this research. XL performed most experiments and analyzed all the data. CL performed the GUS staining assay. SH provided the plant materials. XW, DC, and JS participated in the experiments. MW and XL wrote the manuscript. MW and WZ were involved in the data discussions. All authors read and approved the final manuscript.

## Conflict of Interest

The authors declare that the research was conducted in the absence of any commercial or financial relationships that could be construed as a potential conflict of interest.

## Footnotes

1 phosphat.uni-hohenheim.de/

2 http://www.premierbiosoft.com/index.html
