# Supplementary material for: The LRR-RLK Protein HSL3 Regulates Stomatal Closure and the Drought Stress Response by Modulating Hydrogen Peroxide Homeostasis
Source: Front Plant Sci. 2020 Nov 27;11:548034. doi: 10.3389/fpls.2020.548034 (PMC7728693; doi:10.3389/fpls.2020.548034)
Supplement: Supplementary file 1 [file Table_1.doc]

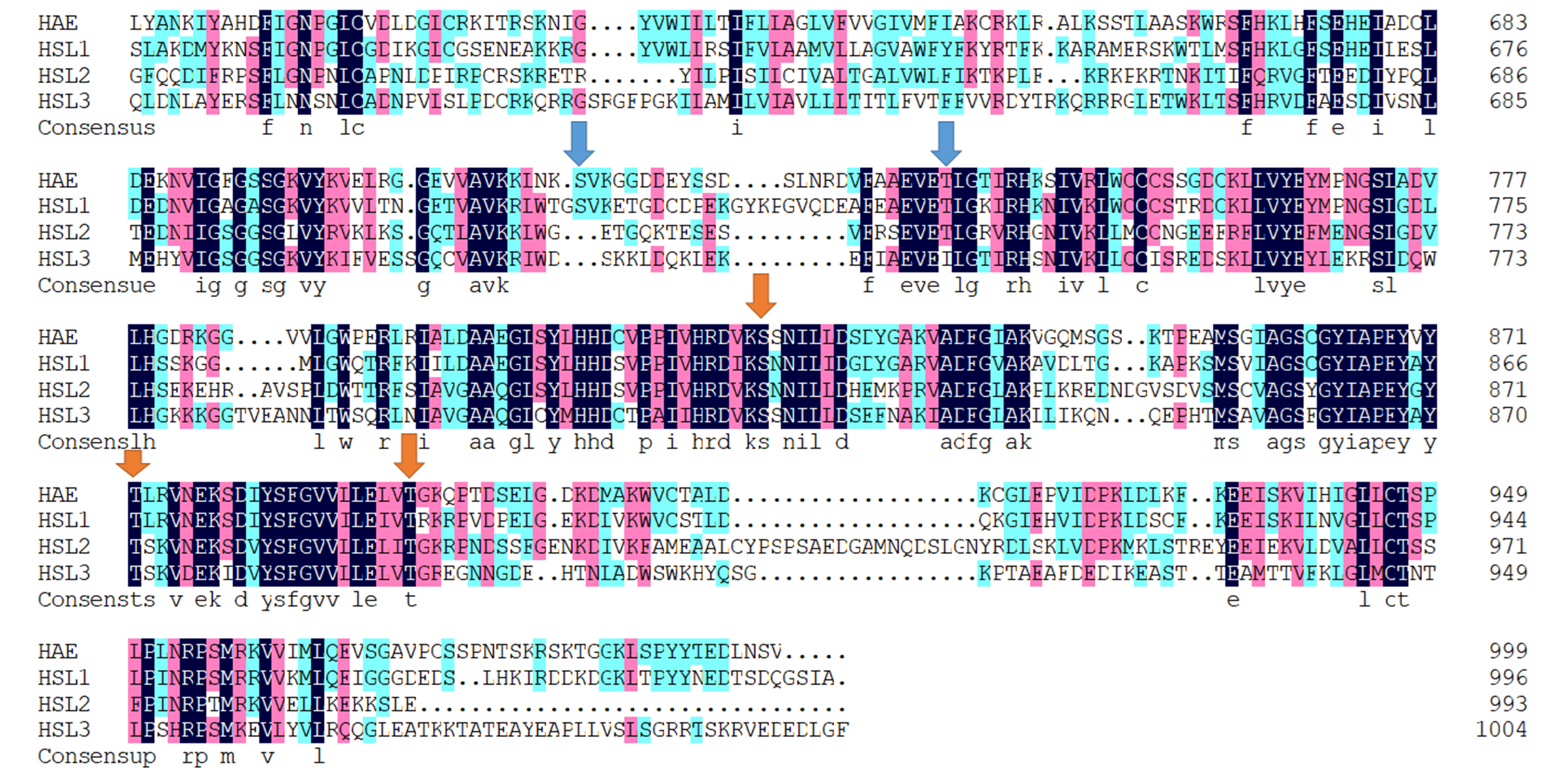


**Supplementary Fig. S1** Amino acid sequence alignment of the kinase domain among HSL3, HSL2, HSL1 and HAE. Conserved phosphorylation sites in this protein family were indicated by arrows. The orange arrows indicated the phosphorylation sites were conserved in all of the aligned protein sequences, and the blue arrows indicated the phosphorylation sites were only conserved in HSL2, HSL1 and HAE proteins.


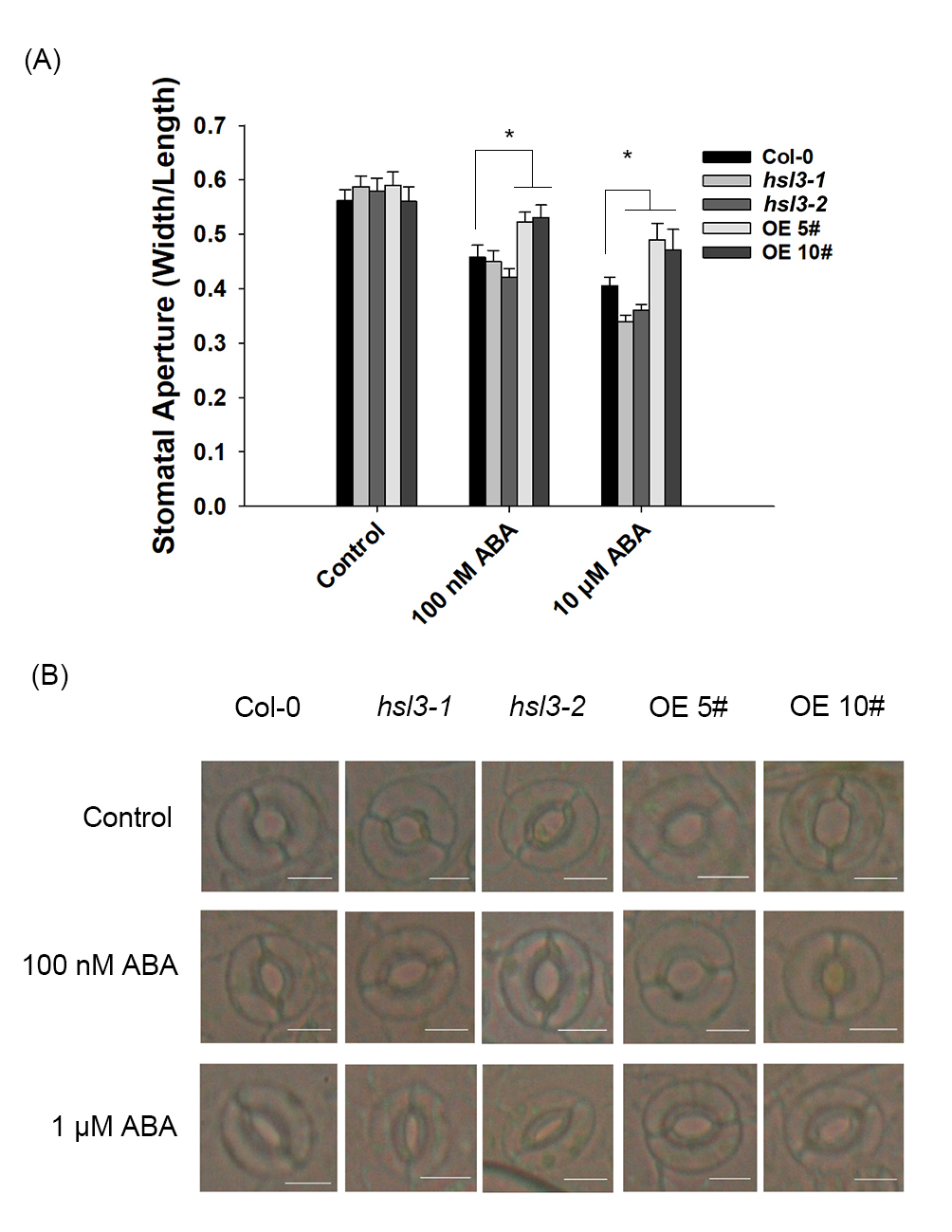


**Supplementary Fig. S2** ABA effect on stomatal closure in Col-0, *hsl3* mutant and the over-expression plants. The experiments were repeated three times. * indicates significant difference from one another at P < 0.05. All data represent mean ±SE (*n*=40). Bar=10 μm.


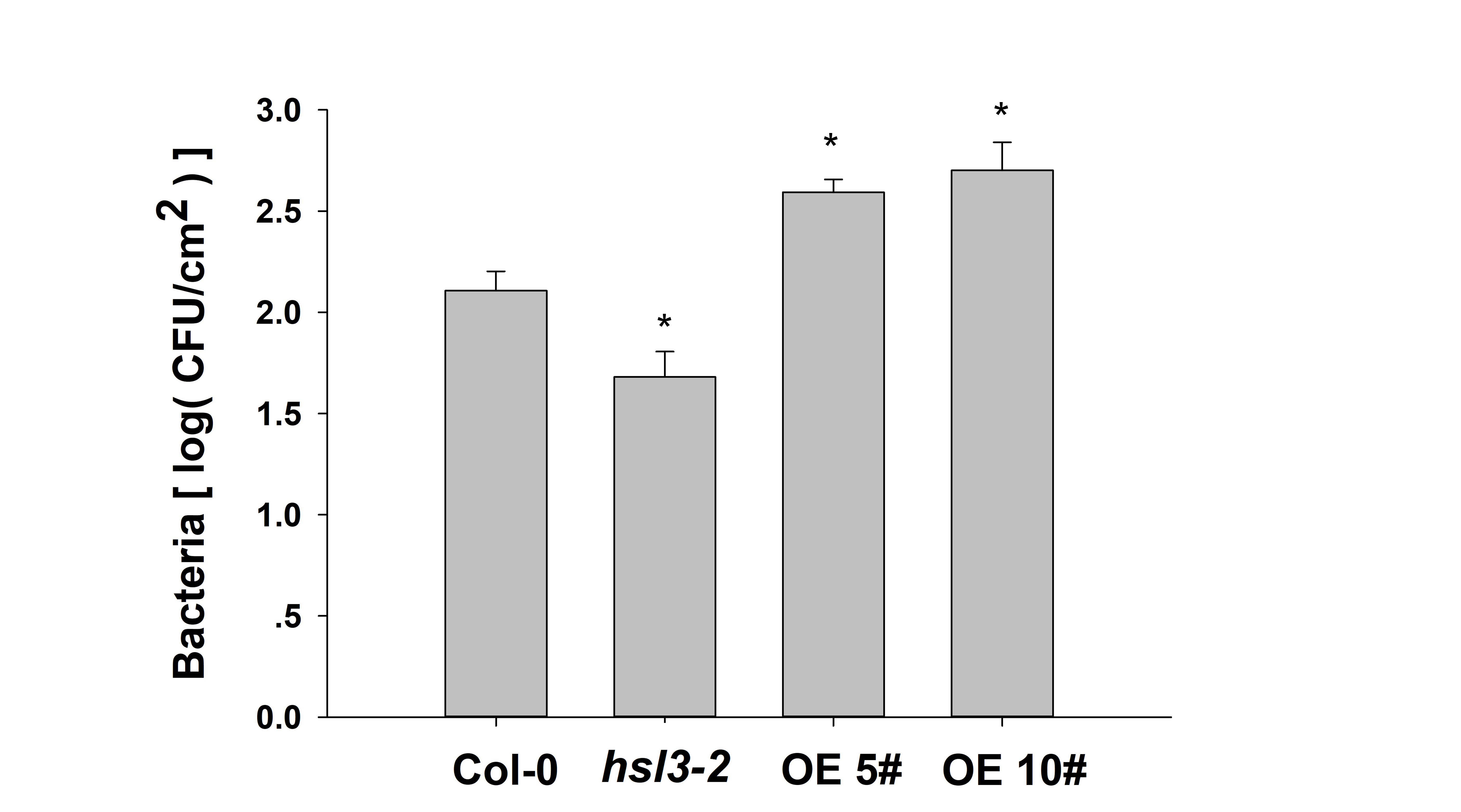


**Supplementary Fig. S3** HLS3 promotes the susceptibility to *Pseudomonas syringae* pv *tomato* (*Pst*) DC3118. Bacteria growth in Col-0, T-DNA insertion mutant *hsl3-2* and two over-expression (OE) lines 5# and 10#. * indicates significant difference from one another at P < 0.05.
